# β-hydroxybutyrate induces endoplasmic reticulum stress in bovine cumulus cells during oocyte maturation *in vitro*

**DOI:** 10.1590/1984-3143-AR2024-0106

**Published:** 2025-05-30

**Authors:** Daniele Missio, Julia Koch, Zigomar da Silva, Francielli Weber Santos Cibin, Marcos Henrique Barreta, Vitor Braga Rissi, André Lucio Fontana Goetten, Fernando Silveira Mesquita, Valério Marques Portela, Paulo Bayard Dias Gonçalves, Rogério Ferreira

**Affiliations:** 1 Laboratorio de Biotecnologia e Reprodução Animal – BioRep, Universidade Federal de Santa Maria – UFSM, Santa Maria, RS, Brasil; 2 Laboratorio de Biotecnologia da Reprodução – BIOTECH, Universidade Federal do Pampa – Unipampa, Uruguaiana, RS, Brasil; 3 Laboratorio de Fisiologia e Reprodução Animal – LAFRA, Universidade Federal de Santa Catarina – UFSC, Curitibanos, SC, Brasil; 4 Laboratório de Fisiologia Molecular e Integrativa da Reprodução – MINT, Universidade Federal do Pampa, Uruguaiana, RS, Brasil; 5 Departamento de Fisiologia e Farmacologia, Universidade Federal de Santa Maria – UFSM, Santa Maria, RS, Brasil; 6 Departamento de Zootecnia, Universidade do Estado de Santa Catarina – UDESC, Chapecó, SC, Brasil

**Keywords:** β-hydroxybutyrate, cattle, ketose, negative energy balance, oocyte

## Abstract

Ketosis is a significant metabolic disorder caused by negative energy balance (NEB). β-hydroxybutyrate (BHBA) is the most abundant circulating ketone body and is known to accumulate within follicular fluid. Although NEB and metabolic stress conditions have been associated with reduced fertility in cows, the effect of increased BHBA on the viability and functionality of bovine cumulus-oocyte complexes (COC) is poorly understood. The aim of this study was to determine the effects of BHBA on expansion, oxidative status, and endoplasmic reticulum (ER) stress in cumulus cells, oocyte nuclear maturation, and *in vitro* embryo production (IVP) in cattle. Cumulus-oocyte complexes were treated with 0, 2, or 4 mM of BHBA for 6, 12, 18, or 24 h during oocyte *in vitro* maturation (IVM.) Cumulus expansion and mRNA levels of genes related to cumulus expansion, oxidative status, ER stress, and autophagy were evaluated. The oocytes were fixed for nuclear maturation analysis at 24 h of IVM. In addition, after IVM with BHBA for 24 hours, cleavage and blastocyst rates were examined. No difference was observed in the rates of oocytes reaching metaphase II, cleavage, and blastocysts. Furthermore, the oxidative status and relative abundance of mRNA for genes associated with antioxidant enzymes and autophagy were not altered by BHBA in cumulus cells. However, BHBA altered the total area of the COC and increased the mRNA levels of genes associated with ER. In conclusion, BHBA affects the expansion and induces ER stress in bovine cumulus cells during IVM without compromising oocyte nuclear maturation, oxidative status, cleavage, and blastocyst rates.

## Introduction

Metabolic stress conditions leading to negative energy balance (NEB) have been associated with reduced fertility in cows (Raboisson et al., 2014; Roche et al., 2017). The period of NEB coincides with the resumption of ovarian activity during the postpartum period (Roche et al., 2017). In this period, concentrations of non-esterified fatty acids (NEFA) and ketone bodies are elevated, while blood glucose concentrations are reduced in the blood plasma and reflected in the follicular fluid (Leroy et al., 2005; Lin et al., 2012; Aardema et al., 2013; Valckx et al., 2014). Additionally, studies have reported that postpartum ovulation rates are lower in cows with higher serum concentrations of ketone bodies (Hill et al., 2018; Stevenson et al., 2020).

Beta-hydroxybutyrate (BHBA) is the most abundant circulating ketone body, and its concentration is a marker of fatty acid oxidation. The BHBA increases considerably during fasting, prolonged exercise, or diabetic ketoacidosis in humans and ketosis in cattle (Newman and Verdin, 2014). Dairy cows with BHBA plasma concentrations above 1.4 mM are considered to be in ketosis (Duffield et al., 2009). In cases of persistent nutritional imbalance, cows develop severe clinical ketosis (plasma BHBA > 3 mM; Foster, 1988). There is a strong correlation between the concentration of BHBA in the serum and follicular fluid (Leroy et al., 2004). The follicle is the microenvironment where cumulus-oocyte complexes (COC) develop and mature (Leroy et al., 2015). Therefore, in ketotic cows, cumulus cells are exposed to high concentrations of this endogenous metabolite (Sangalli et al., 2018).

Throughout follicular development, the oocyte and cumulus cells communicate via a bidirectional pathway (Kidder and Vanderhyden, 2010). Cumulus cells play several critical roles during oocyte development and maturation, including the exchange of molecules that promote meiotic resumption, protection against oxidative stress, oocyte molecular and cytoplasmic maturation, and ovulation (Tanghe et al., 2002; Shimada et al., 2006). Thus, the cumulus cell layer may be considered, after the blood-follicle barrier, the second barrier between blood and the oocyte (Aardema et al., 2019). Studies have shown that during the maturation stage, cumulus cells appear to effectively protect oocytes against stress caused by fatty acids in cattle (Aardema et al., 2013) or environmental contaminants in rats (Campen et al., 2017).

Ketone bodies, including BHBA, increase the production of reactive oxygen species (ROS; Hawkins and Biebuyck, 1979) and promote oxidative stress (Youssef et al., 2010; Wullepit et al., 2012; Song et al., 2014). Oxidative stress has been proven to be an initiator and one of the main contributors to endoplasmic reticulum (ER) stress (Hotamisligil, 2010). ER stress is a cytoprotective mechanism activated under non physiological conditions (Fernández et al., 2015), such as ketosis in dairy cows. However, to our knowledge, no studies are available on the isolated and direct effects of BHBA on bovine cumulus cells. Although cows experience elevated concentrations of NEFA and BHBA, during NEB, this study focused on understanding the direct impact of BHBA in cumulus cells and oocyte, without the concomitant presence of NEFA, to elucidate the specific mechanisms related to this ketone body. Therefore, the aim of our study was to determine the effects of BHBA on cumulus expansion, oxidative status, ER stress, and autophagy in cumulus cells, oocyte nuclear maturation and embryo development in cattle.

## Methods

All chemicals used in this study were purchased from Sigma Chemicals Company (St. Louis, MO, USA), unless otherwise stated. The research does not require ethics committee approval.

### Experimental design

To investigate the effect of BHBA on bovine cumulus cells, primarily COC with compact, multilayered cumulus and homogeneous ooplasm (grade 1 COC; (Leibfried and First, 1979) were randomly allocated into Control, 2, or 4 mM of BHBA groups (*n* = 5 COC/group/repeated four times) and cultured in 200 μL of maturation medium. After 6, 12, 18, and 24 hours of *in vitro* maturation, COC area was evaluated, and subsequently, the cumulus cells were removed by pipetting. While cumulus cells were used for the evaluation of gene expression and the oocytes were used for nuclear maturation assessment. Posteriorly, to investigate the effect of BHBA on the oxidative status of cumulus cells (*n* = 45 to 50 COC/group/repeated four times), grade 1 COC were randomly divided into groups and cultured in 400 μL of maturation medium cultivated by 12 or 24 hours. Cumulus cells were removed and frozen to evaluate ROS production and total antioxidant capacity. To *in vitro* embryo production, grade 1 COC (*n* = 35 to 45 COC/ group/repeated three times) were randomly divided into groups 0, 2 and 4 mM de BHBA and cultured in 400 μL of IVM for 24 hours. After IVF, probable zygotes were cultured for 7 days to evaluate cleavage and blastocyst rates, and embryo cell number. The concentrations of 0 (control), 2, or 4 mM of BHBA used in this study were based on concentrations measured in animals suffering from subclinical and clinical ketosis (Itle et al., 2015).

### Oocyte recovery and *in vitro* maturation

Ovaries were obtained from cows at different stages of the estrous cycle in an abattoir and transported to the laboratory in saline solution (0.9% NaCl) at 30 °C, containing 100 IU/mL penicillin and 50 μg/mL streptomycin sulphate. The COC were aspirated from 3 to 8 mm ovarian follicles. Only grade 1 COC were recovered and selected under a stereomicroscope according to Leibfried and First (1979). Subsequently, the COC were transferred to four-well culture dishes (Nunc®, Roskilde, Denmark) with maturation medium and the components of each treatment. The basic maturation medium used was Medium 199 (1X) containing Earle’s salts, L-glutamine, 2.2 mg/mL sodium bicarbonate and 25 mM Hepes (Gibco Labs, Grand Island, NY, USA), supplemented with 0.2 mM pyruvic acid, 5.0 µg/mL LH (Bioniche, Belleville, ON, Canada), 0.5 µg/mL of FSH (Folltropin®-V, Bioniche, ON, CA), 0.4% (v/v) bovine serum albumin (BSA), 100 IU/mL penicillin, and 50 μg/mL streptomycin sulphate. Then, the COC were cultured at 38.5 °C in an atmosphere containing 5% CO_2_ in air, at 95% relative humidity, for 6, 12, 18, or 24 h.

### *In vitro* fertilization (IVF) and *in vitro* embryo culture (IVC)

To evaluate the potential deleterious effect of BHBA on *in vitro* embryo production, bovine oocytes were fertilized *in vitro* with frozen thawed semen separated by a discontinuous Percoll (GE Healthcare, São Paulo, SP, Brazil) gradient after IVM. Sperm was diluted and added to the COC plate at a final adjusted concentration of 2 × 10^6^ sperm/mL in Fert TALP medium containing 10 μg/mL heparin, 30 μg/mL penicillamine, 15 μM hypotaurine, and 1 μM epinephrine (Parrish et al., 1986), 100 IU/mL penicillin, and 50 µg/mL streptomycin sulfate. Fertilization was carried out by co-culturing of sperm and oocytes for 18 to 22 hours in four well plates (Nunc, Roskilde, Denmark) under the same atmospheric conditions used for maturation. The day of IVF was considered Day 0 of embryo production.

After IVF, presumptive bovine zygotes were denuded by 2 minutes of vortexing and then cultured in groups of 35 to 45 in a culture chamber (CBS Scientific, Del Mar, CA) at 38.5 °C and a saturated humidity atmosphere of 5% CO_2_, 5% O_2_, and 90% N_2_ in 400 µL of synthetic oviduct fluid medium (SOFaaci ; Holm et al., 1999) containing 0.2 mM pyruvic acid, 5% fetal bovine serum (FBS), 100 IU/mL penicillin, and 50 µg/mL streptomycin sulfate in four well plates. Cleavage rates were evaluated 48 hours post-insemination (D2), and blastocyst rates were assessed on D7 post-insemination. *In vitro* embryo production was performed in three replicates. For the *in vitro* embryo analysis, a control group of COC matured with 10% of FBS instead of BSA was added.

### Cumulus expansion evaluation

Cumulus-oocyte complex expansion was measured at 0, 6, 12, 18, and 24 hours of IVM. Digital images of the COCs were captured through the Leica Application Suite (LAS, Version 3.8) software at 100× magnification. Using the obtained images, the total COC area was analyzed using ImageJ software (version 1.47, National Institutes of Health, Bethesda, MD, USA) at different times and treatments with BHBA.

### Assessment of oocyte nuclear maturation

To analyze the effect of BHBA treatment on nuclear maturation, after 24 hours of IVM, the cumulus cells were removed by repeated pipetting, and denuded oocytes were fixed in 4% paraformaldehyde for 15 minutes, followed by permeabilization of the nuclear membranes with 0.5% Triton X-100 until evaluation. For the assessment of the nuclear maturation phase, the oocytes were exposed to 10 µg/mL of bisbenzimide (Hoescht 33342) for 15 minutes. Stained oocytes were assessed under UV light (wavelength of 340 a 380 nm) using a fluorescence microscope (Leica DMI4000B, Wetzlar, Germany) and considered mature if they displayed a chromatin configuration corresponding to the metaphase II stage.

### Evaluation of oxidative status in cumulus cells

To assess the oxidative status, samples of cumulus cells were subjected to two evaluations: reactive oxygen species (ROS) production and total antioxidant capacity at 0, 12, and 24 hours of IVM. The ROS production was determined using a spectrofluorimetric method according to Loetchutinat et al. (2005). Briefly, the samples were incubated in the dark with 5 µL of 2′,7′ dichloro dihydrofluorescein diacetate (DCHF-DA). The DCHF-DA, upon oxidation, converts to the fluorescent 2′,7′dichlorofluorescein (DCF). The oxidation of DCHF-DA to DCF was used to detect and measure intracellular ROS concentrations. The fluorescence intensity emitted at 520 nm (excitation at 488 nm) was monitored 60 minutes after the addition of DCHF-DA. The total antioxidant capacity was determined by the ferric reducing antioxidant power (FRAP) assay. In the FRAP assay, the antioxidant potential was evaluated by measuring the conversion/reduction of Fe^+3^ to Fe^+2^, which is chelated by 2,4,6-tri(2-pyridyl)-striazine (TPTZ) to form Fe^+2^ -TPTZ complex, with a maximum absorbance at 593 nm, according to Benzie and Strain (1996).

### RNA isolation, reverse transcription, and quantitative real-time PCR

Cumulus cells were removed by repeated pipetting and immediately stored in TRIzol^®^, with the objective of evaluating how BHBA treatment influences cumulus expansion, oxidative status, ER stress, and autophagy. Total RNA was extracted using TRIzol^®^ according to the manufacturer’s instructions and quantified at a wavelength of 260 nm a spectrophotometer (NanoDrop1000, Thermo Scientific, Wilmington, DE, USA). Total RNA (200 ng) was first treated with 0.2 μL DNAse (Invitrogen, Carlsbad, CA, USA) at 27 °C for 15 minutes to digest any contaminating genomic DNA. This was followed by the addition of 0.2 μL EDTA (Invitrogen, Carlsbad, CA, USA) and heating to 65 °C for 10 minutes to stop the reaction. RNA was reverse transcribed (RT) with 1 μL iScript^™^ cDNA Synthesis Kit (Bio-Rad, Des Plaines, IL, USA) at 25 °C for 5 minutes and 46 °C for 30 minutes. The reaction was ended by incubation at 95 °C for 5 minutes.

Quantitative real-time PCR was performed using a CFX384 thermocycler (BioRad) with BRYT Green® dye and Taq DNA polymerase from GoTaq® qPCR Master Mix (Promega Corporation) along with specific primers (Table 1). Standard two-step qPCR was performed with an initial denaturation step at 95 °C for 3 minutes, followed by 40 cycles of denaturation step at 95 °C for 10 seconds and annealing/extension at 60 °C for 1 minute. Reactions were performed in duplicate, and the melting curve was analyzed to determine the specificity of amplification. The target mRNA concentration was normalized to the amplification of the reference genes glyceraldehyde-3-phosphate dehydrogenase (*GAPDH),* ribosomal protein S18 (*RPS18),* and peptidylprolyl isomerase A (*PPIA)*. Relative expression calculation was performed as described by Pfaffl (2001).

**Table 1:** List of primer used in the qRT-PCR reactions.

**Gene name**	**Sequence (5’ to 3’)**	**Accession number**
***GAPDH***	F: GATTGTCAGCAATGCCTCCT	NM_001034034.2
	R: GTCATAAGTCCCTCCACGA	
***PPIA***	F: GGTCATCGGTCTCTTTGGAA	NM_178320.2
	R: TCCTTGATCACACGATGGAA	
***RPS18***	F: CCTTCCGCGAGGATCCATTG	NC_037350.1
	R: CGCTCCCAAGATCCAACTAC	
***SOD1***	F: ATACACAAGGCTGTACCAGTGC	NM_174615.2
	R: CACATTGCCCAGGTCTCCAA	
***CAT***	F: AGAGGAAACGCCTGTGTGAG	NM_001035386.1
	R: ATGCGGGAGCCATATTCAGG	
***GPX1***	F: GCATCAGGAAAACGCCAAGA	NM_174076.3
	R: CCATTCACCTCGCACTTTTCG	
***HSPA5***	F: CGTGCGTTTGAGAGCTCAGT	NM_001075148.1
	R: GACAGCTTCATCTTTCCAGCG	
***XBP1u***	F: GCAGAGACCAAGGGGAATGG	NM_001271737.1
	R:GGGTCCAAGTTGAACAGAATGC	
***XBP1s***	F: AGCAGAGACCAAGGGGAATG	NM_001034727.3
	R: TCAGAGTCCATGGGGAGATGT	
***ATF6***	F: GAACTTCGAGGATGGGTTCATAGG	NC_037330.1
	R: CCAGAGCACCCTGAAGAATACG	
***CHOP***	F: GGTGCTGTCCTCAGATGAAAATCG	NM_001078163.1
	R: GGTCCTGGCTCCTCAGTAAGC	
***LC3***	F: GCCGAACCTTCGAACAAAGAG	NC_037345.1
	R: TGAGCTGTAAGCGCCTTCTT	
***HAS2***	F: GCATGTCACCCAGTTGGTCT	NC_037341.1
	R: TGGGTCAAGCATGGTGTCTG	
***TNFAIP6***	F: GCTCACGGATGGGGATTCAA	NC_037329.1
	R: CGTGCTTCCCTGTGGTAGAC	

F: Forward primer; R: Reverse primer.

### Estimate of the total cell number after embryo culture

After 7 days in culture, the embryos were rinsed in PBS containing 0.1% polyvinyl alcohol (PVA) and fixed for 15 to 20 minutes in 4% paraformaldehyde. The fixed embryos were then rinsed in PBS containing 0.1% PVA and stored at 4 °C in PBS supplemented with 0.5% Triton X-100 and 0.1% PVA for 1 hour. Two to three embryos were placed on to each slide containing 10 μM Hoechst 33342 for 10-15 minutes. The embryo samples were mounted on glass slides with Mowiol. The blastocysts were examined and documented using a stereomicroscope (BX53, Olympus, Japan) equipped with a DP73 camera (Olympus, Tokyo, Japan). The total number of blastocyst nuclei was analyzed using ImageJ software (version 1.47, National Institutes of Health, Bethesda, MD, USA).

### Statistical analyses

Statistical analyses were performed using JMP software (JMP Statistical Discovery LLC, Cary, NC, USA). Continuous data were tested for normal distribution using the Shapiro-Wilk test and normalized, when necessary, based on the data distribution and residuals of each statistical model. The effect of treatments on the COC area over time was analyzed using mixed models for repeated data, with each COC treated as a subject. Different covariance structures were tested for each model, and the one with the lowest Akaike Information Criteria (AIC) was selected. Differences in COC diameter at specific time points were compared using Tukey's honestly significant difference (HSD) test. Other continuous dependent variables were subjected to one-way ANOVA, followed by the and Tukey HSD test as a *post-hoc analysis.* A significant level of *P* < 0.05 was considered statistically significant. Data are presented as least square corrected means + SEM (standard error of the mean).

## Results

**Figure 1:**
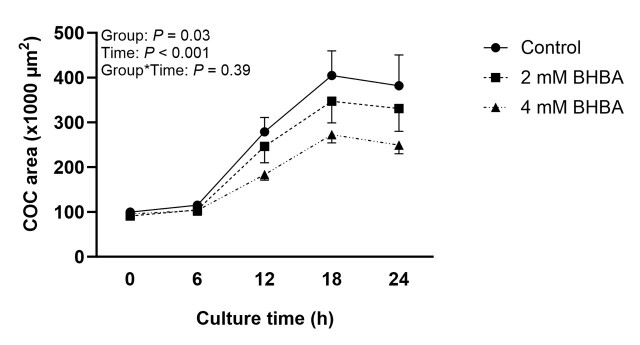
Total area of bovine COC at 0, 6, 12, 18, and 24 hours of oocyte *in vitro* maturation (IVM) without (control) or with 2 or 4 mM of β-hydroxybutyrate (BHBA). Data are presented as mean ± SEM of four replications.

The total area of the COC was measured at 0, 6, 12, 18, and 24 hours of IVM. Our results demonstrate that there was no interaction between BHBA treatment and different times (*P* = 0.39) during IVM. However, there was a treatment effect on the total COC area (*P* = 0.03). The 4 mM BHBA treatment reduced the overall COC area (160.20 ± 1.06 x 1000 µm^2^) compared to the Control (199.53 ± 1.05 x 1000 µm^2^; *P* < 0.05), and neither group differed from the 2 mM group (178.68 ± 1.06 x 1000 µm^2^; Figure 1). Regarding the effect of BHBA on the stage of meiotic progression, the number of oocytes incubated in the presence of 2 mM or 4 mM BHBA that reached metaphase II (81.9 ± 2.11%; 82.6 ± 2.11%, respectively) in 24 hours was not different from that of the control group (84 ± 1.79%; *P* = 0.72).

**Figure 2:**
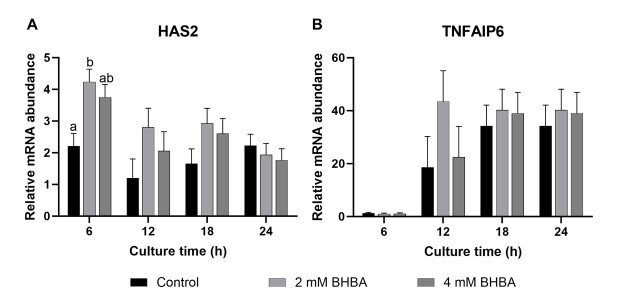
Messenger RNA abundance of hyaluronan synthase 2 (*HAS2;* A) and tumor necrosis factor alpha-induced protein 6 (*TNFAIP6;* B) in cumulus cells after cumulus-oocyte complexes *in vitro* maturation without (control) or with 2 or 4 mM of β-hydroxybutyrate (BHBA). Data are presented as mean ± SEM of four replications. Different letters at the same time point indicates statistical difference (*P* < 0.05).

In this study, the transcript abundance of cumulus expansion-related genes (hyaluronan synthase 2 - *HAS2* and tumor necrosis factor alpha-induced protein 6 - *TNFAIP6*) was examined at 6, 12, 18, and 24 hours of IVM. BHBA at 2- or 4-mM concentrations did not affect the expression of *HAS2* or *TNFAIP6* mRNA abundance at the different times examined, except for *HAS2,* which was induced by 2 mM of BHBA at 6 hours of COC maturation (*P* < 0.01; Figure 2A and 2B).

**Figure 3:**
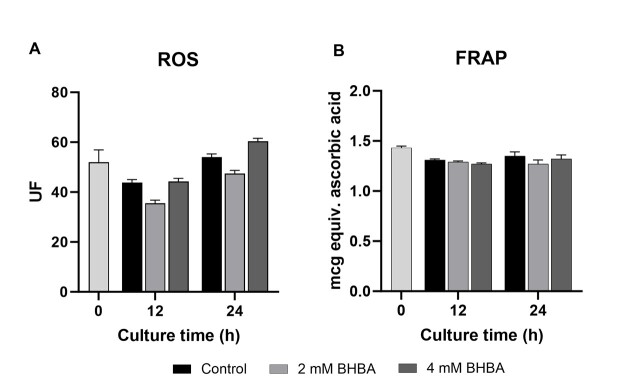
Reactive oxygen species (ROS; A) and ferric reducing antioxidant power (FRAP; B) in cumulus cells after maturation of cumulus-oocyte complexes (COC) without (control) or with 2 or 4 mM of β-hydroxybutyrate (BHBA). Data are presented as mean ± SEM of four replications (*P* > 0.05).

In this study, the transcript abundance of the antioxidant enzymes Cu/Zn superoxide dismutase (SOD1), catalase (CAT), and glutathione peroxidase (GPX1) was evaluated. The *SOD1, CAT,* and *GPX1* mRNA abundance in cumulus cells was not affected by 2 or 4 mM of BHBA during oocyte maturation at different times examined. To further explore the oxidative status of cumulus cells exposed to BHBA, we evaluated ROS production and total antioxidant capacity at 12 and 24 hours of IVM. None of these variables were affected by the BHBA treatment (2 or 4 mM; Figure 3; *P* > 0.05).

**Figure 4:**
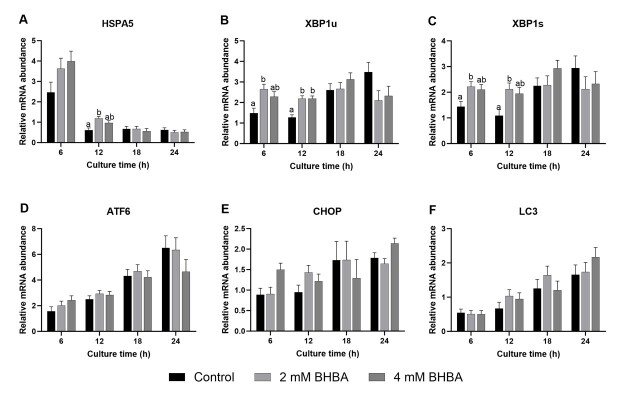
Messenger RNA abundance of heat shock 70 kDa protein 5 (*HSPA5;A*), X-box binding protein 1 unspliced (*XBP1u; B*), X-box binding protein 1 spliced (*XBP1s;C*), activating transcription factor 6 (*ATF6; D*), DNA damage inducible transcript 3 (*CHOP; E*), and microtubule-associated protein 1A/1B-light chain 3 (*LC3; F*) in cumulus cells after maturation of cumulus-oocyte complexes (COC) without (control) or with 2 or 4 mM of β-hydroxybutyrate (BHBA). Data are presented as mean ± SEM of four replications. Different letters represent statistical difference (*P* < 0.05).

The abundance of five ER stress marker transcripts (heat shock 70 kDa protein 5 - *HSPA5*, activating transcription factor 6 - *ATF6*, X-box binding protein 1 spliced - *XBP1s*, X-box binding protein 1 unspliced - *XBP1u*, and DNA damage-inducible transcript 3 - *CHOP)* was evaluated (Figure 4). *HSPA5*, *XBP1s,* and *XBP1u* mRNA levels increased in cumulus cells when 2 mM BHBA was present in the maturation medium for 6 and/or 12 h, but this increase was not observed at any other time points (Figure 4, *P* < 0.05). The mRNA abundance of the other ER stress marker transcripts examined did not differ from that of control cumulus cells (Figure 4, *P* > 0.05). Additionally, in response to oxidative and ER stress, cells can trigger an autophagic response to reduce their damage. Thus, we examined microtubule-associated protein 1A/1B-light chain 3 (*LC3*, the main autophagic marker) gene expression in bovine cumulus cells exposed to different BHBA concentrations. BHBA did not alter *LC3* mRNA levels compared to those cultured in the control group at any of the IVM times (Figure 4F).

**Figure 5:**
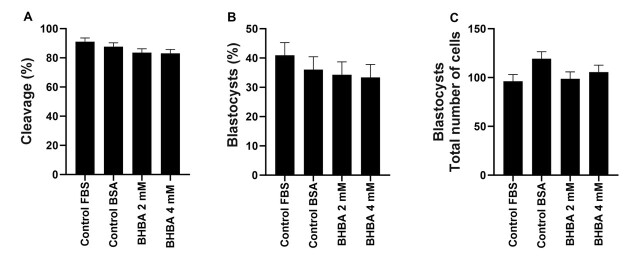
Cleavage rate (A), Blastocysts rate (B) and total number of blastocyst cells (C) of oocytes *in vitro* matured without (control) or with 2 or 4 mM of β-hydroxybutyrate (BHBA). Data are presented as mean ± SEM of three replications (*P* > 0.05). Fetal Bovine Serum (FBS); Bovine Serum Albumin (BSA).

BHBA had no effect on the embryo cleavage rate at concentrations of 2 mM of BHBA (83.6 ± 2.6%) or 4 mM of BHBA (83.2 ± 2.6%) when compared to the control group with FBS (91.07 ± 2.6%) or BSA (87.7 ± 2.6%; *P* = 0.18; Figure 5A). Moreover, 7 days after IVF, the blastocyst rate was similar among the control, 2 mM, and 4 mM groups (*P* = 0.64; Figure 5B), indicating that *in vitro* maturation of oocytes with BHBA had no effect on embryo development. The total number of blastocyst cells (Figure 5C) was similar among the control + FBS (96.15 ± 6.9), control + BSA (119.37 ± 7.07), 2 mM (98.68 ± 7.07), and 4 mM (105.74 ± 6.98) BHBA groups (*P* = 0.09).

## Discussion

In the present study, we investigated the effects of high concentration of BHBA during *in vitro* maturation on cumulus cell expansion, gene expression, oxidative stress status, oocyte maturation, and developmental potential. BHBA exposure during IVM upregulated transcripts associated with ER stress. Additionally, BHBA caused a reduction in COC area and an increase in the abundance of transcripts that regulates cumulus expansion. These results suggest that this ketone body temporarily alters molecular mechanisms and the phenotype of bovine cumulus cells. The observed changes in the transcriptional profile suggest that a stress response mechanism is activated by exposure to BHBA.

The BHBA at 4 mM reduces the overall average COC area. However, at 2 mM BHBA increased *HAS2* mRNA expression at 6 hours of IVM. *TNFAIP6* expression and COC area were not altered. *HAS2* and *TNFAIP6* are necessary for the synthesis of hyaluronic acid (Sugiura et al., 2009) and *HAS2* expression is correlated with oocyte development (Dunning et al., 2007). In our study, no changes were observed regarding oocyte nuclear maturation and cleavage rate during IVM in the presence of BHBA. Another hypothesis is that apoptosis of cumulus cells can be prevented by increasing levels of *HAS2*. Recently, hyaluronic acid and *HAS2* have been reported to protect against apoptosis induced by environmental stress and promote survival in various cell types, including fibroblasts (Wang et al., 2014), granulosa cells (Kaneko et al., 2000; Tunjung et al., 2009), articular chondrocytes (Peng et al., 2010), and human aortic smooth muscle cells (Vigetti et al., 2011). At the same IVM time, the expression of genes related to ER stress (*XBP1s* and *XBP1u*) was elevated in the BHBA groups, and these pathways trigger cellular stress. However, we were unable to elucidate this mechanism in our study, and further studies should be performed to assess it.

β-hydroxybutyrate in the IVM medium of bovine oocytes has a detrimental effect on embryo development, which varies according to the glucose concentration used (Leroy et al., 2006). In this sense, we evaluated whether this BHBA exposure would affect the oocyte’s capacity to reach the metaphase II stage and blastocyst formation. Additionally, the total number of blastocyst cells, which has been associated with the quality and potential of embryonic development, was similar among the groups. β-hydroxybutyrate did not change nuclear maturation, cleavage, and blastocyst rates compared to the control, probably because the cumulus cells protect the oocyte from the effects of BHBA, as observed in the presence of NEFA in cattle (Aardema et al., 2013). The mechanisms of protection can be physical, acting as a barrier between the oocyte and the microenvironment, or molecular, through the metabolism of biomolecules that, in excess, can harm oocyte competence (Dumesic et al., 2015). In agreement with our data, Sangalli et al. (2022, 2018) also observed no difference in the rate of oocytes that reached the metaphase II stage and blastocyst rates after being matured with 6 mM of BHBA. Leroy et al. (2008) did not observe any adverse effects of 4 mM of BHBA on cleavage and embryo production rates in cattle. In swine, BHBA at various concentrations during IVM had no effect on the oocyte’s capacity to reach metaphase II and the cleavage rate (Tsuzuki et al., 2009). It is important to point out that BHBA is not only present during oocyte maturation *in vivo* but also reaches the oocyte during the follicular wave or even at the preantral follicle stage (Leroy et al., 2008). *In vivo*, COCs from cows are exposed to elevated BHBA for a prolonged period during the transition phase and early stages of folliculogenesis (Aardema et al., 2013), which may compromise the functionality of follicular cells and impair reproductive performance. Our group has demonstrated *in vivo* that BHBA does not compromise granulosa cells but does decrease follicular growth and ovulatory follicle diameter in cows (Missio et al., 2022). Moreover, it is known that ovulation from a smaller follicle result in a less competent oocyte (Perry et al., 2005; Read et al., 2021). Therefore, cows in ketosis experience lower follicular growth, leading to altered COC expansion and ER stress in cumulus cells after the LH surge, which compromises their fertility.

Cumulus cells play an important role in oocyte maturation and protect oocytes against cell damage caused by oxidative stress during IVM (Cetica et al., 2001; Combelles et al., 2009). Importantly, oxidative stress is observed in ketotic cows and is positively correlated with high BHBA and NEFA concentrations (Li et al., 2016). Thus, this study examines the effect of BHBA on the oxidative status of bovine cumulus cells by evaluating SOD*1*, *CAT,* and *GPX1* gene expression, ROS production and total antioxidant capacity. According to our results, Sangalli et al. (2018) observed that the abundance of *SOD1, CAT,* and *GPX1* mRNA in early embryo stages did not change when 6 mM of BHBA was present during *in vitro* embryo development. However, BHBA was responsible for increasing antioxidant enzymes activity in other cell types, including renal tissue (Shimazu et al., 2013), spinal cord (Kong et al., 2017) and cardiomyocytes (Nagao et al., 2006) and offers neuroprotection in the treatment or prevention of Alzheimer's and Parkinson's disease in humans (Kashiwaya et al., 2000). Additionally, antioxidant defenses were unable to neutralize ROS in cells other than cumulus cells, leading to oxidative stress, especially when high concentrations of BHBA were used (Shi et al., 2014; Li et al., 2019; Ferst et al., 2021). However, in our study, no difference in oxidative status was observed, which may be attributed to the ability of antioxidant defenses to neutralize ROS. These results, associated with those in the literature, demonstrate the contradictory role of BHBA in cells, both as an inducer or protector of cells from oxidative stress and ER. Moreover, according to Tatemoto et al. (2000) cumulus cells surrounding oocytes during folliculogenesis act as a mechanical barrier that protects oocytes from apoptosis induced by oxidative stress.

Oxidative stress has been shown to be a trigger and one of the main contributors to ER stress (Hotamisligil, 2010). Therefore, we hypothesized that BHBA during IVM induces ER stress in cattle cumulus cells and assessed its effect on ER stress gene responses. In the present study, we examined the abundance of mRNA transcripts involved in the regulation of ER stress (*HSPA5, ATF6, CHOP, XBP1s,* an*d XBP1u;*Kadowaki and Nishitoh, 2013). Unfortunately, we did not assess the abundance or function of proteins in the present study. Our results demonstrate that BHBA increased mRNA levels for *HSPA5*, *XBP1u,* and *XBP1s* genes at 6 and/or 12 hours of IVM. Disturbances in ER homeostasis cause stress, resulting in the accumulation of unfolded proteins in the organelle. The NEB state has been shown to affect the expression of ER stress-related genes and proteins in the liver of starved dairy cows and in bovine mammary epithelial cells cultured *in vitro* for 24 hours (Zhang et al., 2020). Furthermore, recent studies by Shi et al. (2021) and Islam et al. (2021) have demonstrated that ER stress occurs in hepatocytes of cows with ketosis. Activation of unfolded protein response signaling pathways indicates that BHBA increases misfolded protein in the ER lumen in cumulus cells, which may compromise cell viability. However, the detrimental effects of BHBA seemed to be prevented by the activation of cytoprotective mechanisms. Studies have demonstrated the ability of BHBA to mitigate ER in different cell types (Bae et al., 2016; Tagawa et al., 2019). In our IVM results, which did not observe any effects of BHBA on ER stress marker genes in cumulus cells after 12 hours. Studies have indicated that the elevation of NEFA concentration, which is also observed during the NEB period in cows, induces COC endoplasmic reticulum stress in COC during IVM in various species (Yang et al., 2012; Sutton-McDowall et al., 2016; Chen et al., 2019). Consequently, the increase in NEFA and BHBA within the follicular microenvironment during the transition period leads to ER stress in COC, potentially compromising cell viability.

Oxidative and ER stress also contribute to the initiation of autophagy (Yorimitsu et al., 2006). In this study, we examined the expression of the LC3 gene in cumulus cells of COC matured in different BHBA concentrations. Our findings revealed that BHBA did not modify the abundance of the LC3 gene during IVM. The level of intracellular autophagy is often associated with oxidative stress, ER stress, and the balance between cell death and survival (Holczer et al., 2015; Navarro-Yepes et al., 2014). Therefore, our study observed that BHBA did not activate autophagy mechanisms, likely due to its transient effect on the cumulus cells.

## Conclusion

In conclusion, β-hydroxybutyrate induces ER stress in bovine cumulus cells during *in vitro* maturation and affects expansion without compromising oocyte nuclear maturation, oxidative status, and embryo development. Even without directly compromising immediate *in vitro* results, β-hydroxybutyrate may affect oocyte quality and fertility *in vivo*, since this exposure occurs over a long period and was unfortunately not investigated in our study. Investigating the effect of BHBA isolated is essential to understand how this metabolite contributes to cellular responses during the period of NEB in cows. Furthermore, our results provide insights into the mechanisms associated with reduced fertility in cows with ketosis and provide a basis for future studies.
